# Computational structural genomics and clinical evidence suggest BCKDK gain‐of‐function may cause a potentially asymptomatic maple syrup urine disease phenotype

**DOI:** 10.1002/jmd2.12419

**Published:** 2024-04-08

**Authors:** Emily Singh, Young‐In Chi, Jessica Kopesky, Michael Zimmerman, Raul Urrutia, Donald Basel, Jessica Scott Schwoerer

**Affiliations:** ^1^ Division of Genetics, Department of Pediatrics Medical College of Wisconsin Milwaukee Wisconsin USA; ^2^ Mellowes Center for Genomic Sciences and Precision Medicine Medical College of Wisconsin Milwaukee Wisconsin USA; ^3^ Department of Clinical Nutrition Children's Wisconsin Milwaukee Wisconsin USA

**Keywords:** 3D‐genomics, branched‐chain ketoacid dehydrogenase kinase (BCKDK), gain‐of‐function, maple syrup urine disease (MSUD), molecular dynamics simulation, newborn screening

## Abstract

Maple syrup urine disease (MSUD) is a disorder of branched‐chain amino acid metabolism caused by a defect in the branched‐chain α‐ketoacid dehydrogenase (BCKD) complex (OMIM #248600). The hallmark presentation is encephalopathic crisis in neonates, but can also present with metabolic decompensation, developmental delays, and feeding difficulties. Biochemical evidence for MSUD includes elevated branched‐chain amino acids (BCAA) and the pathognomonic presence of alloisoleucine. The BCKD complex contains several subunits associated with autosomal recessive MSUD, while its regulatory proteins have less well‐defined disease associations. We report on two families with the same *BCKDK* variant (c.1115C>G (p.Thr372Arg)). Probands were detected on newborn screening and demonstrated biochemical evidence of MSUD. The variant was identified in reportedly asymptomatic parents and additional family members who had elevated BCAA and alloisoleucine, following an autosomal dominant pattern of inheritance. To better define the functional effect of the variant on the kinase, we completed molecular modeling using sequence‐based (2D), structural‐based (3D), and dynamic‐based (4D) analyses. The *BCKDK* variant modeling indicated a gain‐of‐function which leads to impaired BCAA catabolism consistent with the biochemical evidence in this cohort. Combining the evidence gained from molecular modeling with the absence of metabolic decompensation in our patients and several adult family members, despite encountering stressors typically problematic in classic MSUD, we suggest that heterozygous gain‐of‐function variants in *BCKDK* may represent a novel biochemical phenotype of MSUD with a benign clinical course.

**Synopsis**

Based on clinical history, family studies, and molecular modeling, evidence suggests a heterozygous gain‐of‐function variant in *BCKDK* may lead to an autosomal dominant biochemical phenotype of MSUD with an asymptomatic presentation.

## 1 INTRODUCTION

The breakdown of the branched‐chain amino acids (BCAAs) leucine, isoleucine, and valine for energy production and lipid and protein biosynthesis is a complex process mediated by the branched‐chain α‐ketoacid dehydrogenase (BCKD) complex in the mitochondrial matrix.^1^ Catabolism of BCAA by the BCKD complex involves four subunits: E1α (2‐oxoisovalerate dehydrogenase subunit alpha, mitochondrial, encoded by *BCKDHA*); E1β (2‐oxoisovalerate dehydrogenase subunit beta, mitochondrial, encoded by *BCKDHB*); E2 (lipoamide acyltransferase component of BCKD complex, mitochondrial, encoded by *DBT*); and E3 (dihydrolipoamide dehydrogenase, mitochondrial, encoded by *DLD*).^2^ Activity of the BCKD complex is tightly regulated by protein phosphatase Mn(2+)‐dependent 1 K (encoded by *PPM1K*), which up‐regulates BCKD complex activity through dephosphorylation of the E1 α subunit, and branched‐chain ketoacid dehydrogenase kinase (encoded by *BCKDK*), which down‐regulates BCKD complex activity through phosphorylation of the S293 residue of the E1 subunit of the BCKD complex while tethering onto the E2 subunit tail through binding to its lipoyl group.^3^ *PPM1K* loss‐of‐function would be expected to result in increased BCAA levels, while gain‐of‐function would be expected to result in decreased BCAA levels. *BCKDK* loss‐of‐function would be expected to result in decreased BCAA levels, while gain‐of‐function would be expected to result in increased BCAA levels.

Dysregulation of the BCAA catabolic pathway can result in a number of human disease presentations. High BCAA levels due to a defect in subunits E1α, E1β, or E2 of the BCKD complex causes classic autosomal recessive maple syrup urine disease (MSUD) (OMIM 248600).^4^ In the most severe cases of MSUD, presentation often occurs within the first week of life with poor feeding, lethargy, abnormal movements, and eventually coma. Milder individuals may present later in life with poor feeding and growth, developmental delays, or an acute encephalopathic event.^5^ At the mildest end of the disease spectrum, individuals with intermittent MSUD are asymptomatic and often have normal BCAA when well but are at risk for decompensation during times of illness, with concomitant BCAA elevations. The pathophysiology of MSUD is related to the neurotoxicity of leucine, which leads to cerebral edema, depletion of glutamate levels, oxidative stress, competition with other important amino acids within the nervous system, and the accumulation of direct neurotoxic metabolites like α‐ketoisocaproic acid.^6^

In addition to defects in the BCKD complex, functional impacts on its regulatory proteins have been shown to cause biochemical abnormalities, though the clinical impacts are less well understood. Two cases have been reported of individuals with homozygous, predicted loss‐of‐function *PPM1K* variants and mild to moderate elevations in BCAA; both individuals were managed with protein‐restricted diets without obvious MSUD symptomatology, and neither individual has experienced a true metabolic decompensation.^7^, ^8^ Loss‐of‐function of BCKDK, resulting in low BCAA levels due to upregulation of the BCKD complex, has been identified to cause a treatable autosomal recessive neurodevelopmental disorder characterized by intellectual disability, autism, and epilepsy.^4^, ^9^, ^10^ Only one published report has described a heterozygous *BCKDK* gain‐of‐function variant in an infant whose newborn screen showed normal BCAA values, but abnormal ratios, leading to second‐tier testing that revealed elevated alloisoleucine and subsequent clinical plasma amino acids that revealed elevated BCAAs. Both the patient and his father were found to carry the *BCKDK* variant and elevated BCAAs, consistent with autosomal dominant inheritance of an MSUD phenotype, but without MSUD symptomatology or episodes of metabolic decompensation.^11^ Elevated BCAA have also been observed in other metabolic disorders such as diabetes and chronic liver disease and the clinical significance of that elevation is under investigation.^4^, ^12^, ^13^

Most often, MSUD is first suspected due to an abnormal newborn screen. MSUD is a core condition on the recommended uniform screening panel for newborn screening and is screened in all 50 states in the United States.^14^ Typically, it is screened by mass spectrometry on a dried blood spot that shows elevated leucine/isoleucine; second tier alloleucine analysis may also be conducted.^15^ False positives may occur due to supplemental nutrition, including total parental nutrition. The diagnosis of MSUD is made by confirmatory testing in blood and urine. Plasma amino acids show elevated BCAAs and pathognomonic alloisoleucine greater than 5 μmol/L. Urine organic acids reveal branched‐chain amino acid ketoacids, including α‐ketoisocaproic acid (KIC), α‐ketoisovaleric acid, and α‐keto‐β‐methylvaleric acid.^6^ Immediate initiation of dietary therapy can reduce leucine levels, and with long‐term dietary and illness management, individuals can have good growth and developmental outcomes.^2^ The incidence of MSUD is estimated at 1 in 185 000, with an increased incidence in the Mennonite population.^5^

Through newborn screening, we identified two unrelated patients with elevated BCAA and alloisoleucine. Molecular sequencing of genes related to BCAA metabolism revealed the same novel *BCKDK* variant, p.Thr372Arg. Cascade testing identified the variant and elevated BCAA in reportedly healthy adult family members in both cases. Initial comparison of our novel p.Thr372Arg variant to the previously reported p.His162Gln variant suggested that these variants should have similar functional effects. To study the impact of the p.Thr372Arg variant, we performed mechanistic‐based analyses on this variant with known pathogenic or non‐natural loss‐of‐function variants at the B‐K domain interface as controls. We implemented a comprehensive approach that incorporated sequence‐based (2D), structural‐based (3D), and dynamic‐based (4D) analyses to assess the fitness of this abnormal protein. Combined, these analyses point to the gain‐of‐function activity of the p.Thr372Arg variant, which may offer insight into the MSUD phenotype identified in our patients and the patient described by Maguolo et al.^11^

## 2 MATERIALS AND METHODS

### 2.1 Study design

This was an observational report of two unrelated patients undergoing routine clinical care at a single center (Children's Wisconsin, Wisconsin, USA). Study data were collected through a review of electronic medical records from March 2018 to October 2023. Patient data and outcomes are described as known in October 2023. Testing of adult family members comprised familial variant analysis, plasma or dried blood spot amino acids when possible, and limited clinical history assessment by report. Institutional review board approval was not required, but written informed consent was obtained from both families.

### 2.2 Patient samples and analysis

Newborn screening was performed at the Wisconsin State Laboratory of Hygiene (Wisconsin, USA) following routine clinical protocols. Clinical confirmatory tests were performed during inpatient admissions and at outpatient clinical visits. Amino acid analyses were completed on dried blood spot via tandem mass spectrometry performed at the Wisconsin State Laboratory of Hygiene (Wisconsin, USA) or plasma via high‐performance liquid chromatography performed at Children's Wisconsin (Wisconsin, USA). Molecular testing was performed on plasma samples, extracted DNA, or buccal samples at PreventionGenetics (Wisconsin, USA) or Precision Medicine Laboratory (Wisconsin, USA), both Clinical Laboratory Improvement Amendments‐certified, College of American Pathologists‐accredited clinical laboratories. Family member testing was supported through the Medical College of Wisconsin Undiagnosed and Rare Disease Program.

### 2.3 Structure modeling of the human BCKDK catalytic domain and the full‐length in complex with ATP and the bound metal ions

The molecular structure of BCKDK has been highly conserved throughout all mammals.^3^ BCKDK belongs to the histidine kinase family^4^ and is made of the active site‐containing catalytic domain (K domain) and the four‐helix bundle regulatory domain (B‐domain) that contains the lipoyl‐binding site and the allosteric inhibitor (feed‐forward inhibition by α‐ketoisocaproic acid) binding site.^16^, ^17^ Several crystal structures of the BCKDK catalytic domain have been determined in rats, which share a 95.6% sequence identity with the human protein.^18^, ^19^ We used the AlphaFold model structure in which all differential sequences in rats (six residues) have been replaced with the human sequences and the missing regions in the crystal structures, such as the N‐terminal extension and the flexible loop (1–67 and 337–361 in the human sequence), have been completed.^20^ We used two different structures—the catalytic domain only (56–412) and the full‐length protein (1–412)—for our analysis; both structures contain the cofactor ATP and the bound K^+^ and Mg^++^ ions in the active site. We used the functional dimeric structures for our analyses.^19^ For variant structure modeling, amino acid substitutions were made within the Discovery Studio suite version 21.1 (Dassault Systèmes BIOVIA) by mutating the corresponding residues and selecting the side chain rotamer causing the least steric hindrance with the surrounding residues. These structures were subjected to the same energy minimization and molecular dynamics (MD) simulation protocol as the wild type.

### 2.4 Protein folding energy and stability calculation

For impact analysis, we used the functional dimeric models of both the full‐length (1–412) and the kinase domain‐only (56–412) proteins. Notably, we found that both models produced similar findings; thus, we present the data with the kinase domain‐only structures. We used both metrics for structural and dynamic damaging impact of a substitution on the protein effect that are either universal or protein‐specific and derive better mechanistic insights. This set of metrics are structurally and functionally relevant to the molecular fitness of the protein, such as structural perturbation, protein stability, and various time‐dependent molecular interactions.

We assessed the mutational impact on protein stability by the variant‐induced changes in folding energy (ΔΔG_fold_) using FoldX and the pH‐dependent mutation energy protocol implemented in the Discovery Studio suite.^21^, ^22^ We used the energy‐minimized mutant structures for these calculations at pH 7.4 by introducing each substitution to the wild type dimeric structures.

### 2.5 Structure perturbation measurement

We assessed the global and local structure perturbations by measuring the positional displacement of backbone atoms between the entire catalytic domains of the wild type and mutants (global) or only the atoms near the residue of interest between them (local). For local structure perturbation, from the energy‐minimized structures, any residues that reside within a 10 Å radius from the mutation site were selected using PyMol (Molecular Graphics System, Schrödinger, LLC) and calculated for the least‐squared root mean square deviation (RMSD) of the backbone atoms between the wild type and mutants using Coot.^23^ For global structure perturbation, entire backbone atoms were used for RMSD calculation between the structures.

### 2.6 Molecular dynamics simulation and time‐dependent interaction energy calculation

MD simulations were performed using the CHARMm36 all‐atom‐force‐field implemented in the Discovery Studio with a 2‐fs time step.^24^ Molecular models were simulated in a simplified distance‐dependent implicit solvent environment at a dielectric constant of 80 and pH 7.4. Energy minimization commenced for 5000 steps using the steepest decent followed by 5000 steps of conjugate gradient to relax the protein structure that was obtained under the stressed crystal environment. Each system of 10 replicates of the model was independently heated to 300 K over 200 ps and equilibrated for 500 ps followed by a 20 ns production simulation under the constant Number of atoms, Pressure, and Temperature ensemble by changing the initial seed (200 ns total). Structures during the unconstrained dynamics simulation were recorded every 10 ps to give a total of 2000 frames for analyses.

Various time‐dependent molecular interaction free energies were measured using the protocol implemented in the Discovery Studio. This was done using the MD simulation trajectories and by selecting the protein and the interaction groups of interest. Non‐bonded interactions were monitored and dynamic interaction energies (van der Waals and electrostatic energies) for each replicate were calculated from the MD trajectories using the CHARMm36 force‐field and the implicit distance‐dependent dielectric solvent model and averaged.

## 3 RESULTS

### 3.1 Clinical consultation, patient management, biochemical assays, and genetic testing lead to the discovery of a new variant associated with an MSUD phenotype

Patient #1 was born at 33 weeks gestation weighing 1000 g to a 41‐year‐old G6P3 mother by emergency c‐section at an outside hospital. Pregnancy was complicated by chronic hypertension, reversed end‐diastolic flow in the umbilical artery, and intrauterine growth retardation. She was admitted to the neonatal intensive care unit (NICU), where she required nasal prong continuous positive airway pressure for respiratory distress, intravenous (IV) fluids for hypoglycemia, phototherapy for jaundice, and platelet transfusion for thrombocytopenia. At 10 days of age, her fourth newborn screen returned positive for MSUD (Table 1). At that time, she was taking donor human milk fortified to 24 kcal/oz via PO/NG to provide approximately 397 mg/kg of leucine and 4 g/kg of intact protein (Table S1). Her fifth newborn screen showed persistently elevated leucine and alloisoleucine levels consistent with MSUD, at which time she was transferred to our care for the implementation of leucine restricted diet for a presumed diagnosis of MSUD (Table S1). Dietary adjustments were made throughout her admission based on frequent plasma amino acid monitoring and growth. Urine ketones remained negative throughout the admission.

**TABLE 1:** Biochemical, molecular, and clinical data for probands and other family members with *BCKDK* c.1115C>G (p.Thr372Arg) variant.

		Patient #1				Patient #2		Maguolo et al.		
		Patient	Mother	Maternal. Grandfather	Maternal aunt	Patient	Father	Patient	Father	Paternal grandfather
NBS results, μmol/L (ref. range)	Leu/Iso	412^a^ (<305)	N/A	N/A	N/A	340^b^ (<305)	N/A	225^f^ (<250)	N/A	N/A
	Val	256^a^ (<250)				227^b^ (<250)		214 (<250)		
	Allo	8^a^ (0–1)				8^b^ (0–1)		8.2 (<2.0)		
PAAs prior to diet initiation, μmol/L unless otherwise indicated (ref. range)	Leu	420^c^ (50–180)	258 (88–152)	306 (82–190)	242 (82–190)	250 nmol/mL^d^ (48–175) 360^e^ (61–183)	242 (82–190)	428 (75–127)	384	234
	Iso	307^c^ (20–110)	158 (37–77)	177 (38–114)	129 (38–114)	176 nmol/mL^d^ (31–105) 180^e^ (27–80)	113 (38–114)	207 (39–65)	156	111
	Val	505^c^ (80–270)	397 (175–275)	451 (160–332)	363 (160–332)	322 nmol/mL^d^ (83–300) 489^e^ (78–264)	355 (160–332)	418 (158–291)	403	315
	Allo	15^c^ (<5)	31 (0)	16 (0–1)	8 (0–1)	14 nmol/mL^d^ (<2) 18^e^ (0)	16 (0–1)	17 (<2.0)	17	3.6
PAAs in follow‐up, μmol/L unless otherwise indicated (ref. range)	Leu	17–270 (67–157)	N/A	N/A	N/A	14–244 (67–157)	N/A	121–193 (75–127)	N/A	N/A
	Iso	56–302 (32–80)				56–341 (32–80)		63–99 (39–65)		
	Val	101–502 (140–282)				132–704 (140–282)		145–323 (158–291)		
	Allo	8–31 (0)				0–51 (0)		4–11 (<2.0)		
Molecular	*BCKDK*	c.1115C > G (p.Thr372Arg)				c.1115C > G (p.Thr372Arg)		c.486 C > A (p.His162Gln)		
Clinical Information	Current diet	120 mg/kg leu; 1.3 g/kg IP; 1 g/kg MFP	No intentional restriction. Eats meat. 0.7–0.9 g IP/kg	No intentional restriction, eats meat	No intentional restriction, eats meat	93 mg/kg leu; 1.3 g/kg IP; 0.7 g/kg MFP	No intentional restriction. Eats meat. 0.2–0.8 g/kg IP	Diet liberalized with BCAA monitoring	N/A	N/A
	No reported decompensations									
	Stressors	Fever/ intercurrent infections	3 pregnancies and deliveries	Fever/intercurrent infections	1 pregnancy and delivery, several dilations for EoE	Fever/intercurrent infections	Fever/intercurrent infections	Fever/intercurrent infections	N/A	N/A

Abbreviations: Allo, alloisoleucine; EoE, eosinophilic esophagitis; HPLC, high‐performance liquid chromatography; IP, intact protein; Iso, isoleucine; Leu, leucine; MFP, medical food protein; MS/MS, tandem mass spectrometry; NBS, newborn screen; PAAs, plasma amino acids; Val, valine.

^a^ First NBS was inconclusive, second and third NBS were normal, results reflect fourth NBS at DOL 10.

^b^ First NBS was normal, results reflect second NBS at DOL 22.

^c^ Collected at DOL 14 prior to Leu restriction.

^d^ Collected at DOL 27 as repeat NBS prior to leu restriction, MS/MS.

^e^ Collected clinically at DOL 31 prior to leu restriction, HPLC analysis.

^f^ Value includes Leu, Iso, Allo.

**FIGURE 1:**
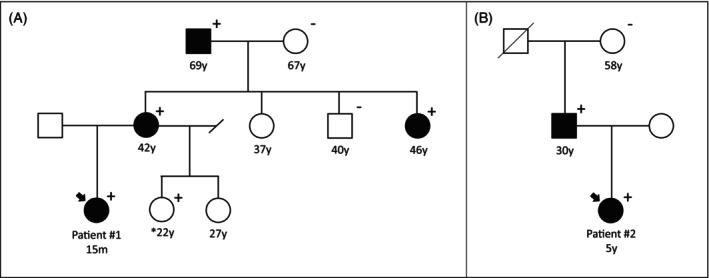
Pedigree of (A) Patient #1 and (B) Patient #2. Plus (+) denotes individuals who carry the *BCKDK* c.1115C>G (p.Thr372Arg) variant. Shaded icons denote individuals with elevated branched‐chain amino acids. *Amino acid analysis was not completed for Patient #1's 22y maternal half‐sister.

Initial next‐generation sequencing of *BCKDHA*, *BCKDHB*, and *DBT* was negative. Subsequent exome sequencing filtered for pediatric‐onset diseases revealed a maternally inherited variant of uncertain significance (VUS) in *BCKDK* (c.1115C>G (p.Thr372Arg)). Subsequent maternal plasma amino acids showed elevations in BCAAs and alloisoleucine (Table 1). Patient #1's mother denied any self‐imposed dietary restrictions, though dietary recall analysis showed typical protein intake near DRI at 0.7–0.9 g/kg/day and a high intake of simple carbohydrates from regular soda. She denied any health concerns typically associated with an MSUD diagnosis. Subsequent family testing identified a maternal aunt and grandfather with the same variant in *BCKDK* and biochemical evidence of MSUD, both of whom denied any health concerns or clinical symptoms typical of MSUD. Pedigree and family health history are outlined in Figure 1 and Table 1.

At 15 months of age (13 months corrected), the patient is demonstrating good catch‐up growth and weight gain. Neurodevelopmental screening on the Capute Scale shows development at or above adjusted age and age‐appropriate motor development by Hammersmith Infant Neurologic Examination. She receives physical therapy monthly as a standard intervention for prematurity. At present, she is tolerating significantly more leucine and intact protein compared to recommendations for classic MSUD patients (approximately 120 mg/kg leucine, 1.3 g/kg intact protein, and 1 g/kg synthetic protein; Table 1) with leucine levels generally near 200 μmol/L (Table S1). Additional diet details are available in Table S1. Based on her own mild presentation thus far and reportedly asymptomatic presentation of family members with the same variant, we plan to begin challenging her intact protein intake as tolerated based on plasma amino acid monitoring and clinical presentation as she enters toddlerhood.

Patient #2 was born at 39 weeks gestation weighing, 3300 g to a 20‐year‐old G2P2 mother by induced, uncomplicated vaginal delivery at an outside hospital. The patient was transferred to the NICU for neonatal abstinence syndrome at 2 days of age and discharged at 24 days of age on a phenobarbital weaning schedule. She was on a combination of breastmilk and standard infant formula throughout admission and after discharge. A repeat newborn screen was obtained at 22 days of life and returned positive for MSUD (Table 1). Elevated leucine and alloisoleucine persisted on a third newborn screen collected at 27 days of age (Table 1), at which time she was referred to our clinic.

The patient was seen at 31 days of age in the metabolic clinic for confirmatory testing and the initiation of an MSUD diet. Confirmatory testing showed an elevated plasma alloisoleucine (Table 1) and normal urine organic acids. Protein‐restricted diet was initially implemented by limiting breastfeeding time throughout the day and supplementing with a combination of MSUD and isovaleric acidemia infant medical foods (Table S1). Plasma amino acids were monitored weekly and dietary adjustments were made based on labs and weight gain to maintain leucine levels in standard MSUD treatment ranges,^25^ though elevated alloisoleucine persisted (Table 1).

Initial sequencing and deletion/duplication analysis of *BCKDHA*, *BCKDHB*, *DBT*, and *DLD* were negative. Additional sequencing and deletion/duplication analysis of *ALDH4A1*, *BCAT1*, *BCAT2*, *PPM1K*, and *PRODH2* was negative shortly thereafter. Due to persistently elevated leucine and alloisoleucine, MSUD management was continued based on a biochemical diagnosis. She has been admitted to the hospital four times for IV dextrose as a precaution in the setting of febrile illnesses with decreased oral intake and has avoided metabolic decompensation at each admission. Leucine levels obtained at or around the time of admission remained within treatment range on the three occasions it was measured, despite febrile illness.

Based on molecular results from Patient #1, *BCKDK* analysis was completed at nearly 5 years of age in Patient #2 and revealed the same VUS as Patient #1, c.1115C>G (p.Thr372Arg). The variant was found to be paternally inherited and subsequent paternal amino acid analysis showed elevated leucine, valine, and alloisoleucine, consistent with MSUD. Patient #2's father denied any self‐imposed dietary restrictions or health concerns typically associated with an MSUD diagnosis, though dietary recall analysis showed a daily range of 0.2–0.8 g/kg intact protein and a high intake of simple carbohydrates from regular soda.

Patient #2 is now 5 years old with normal growth and proportionate weight gain. She received early intervention services for developmental delays, most notably in speech, but she has not required therapies since aging out of the program. A formal neurodevelopmental evaluation has not been completed to date, but no concerns were identified during preschool. Before identifying the *BCKDK* variant, she was maintained on approximately 50 mg/kg leucine, 0.7 g/kg intact protein, and 1 g/kg synthetic protein (Table S1). Since the discovery of the new variant, her modified diet has slowly been liberalized over the past 8 months by increasing her leucine prescription in 10%–20% increments as tolerated based on plasma amino acid monitoring and patient food preferences. At present, she is tolerating approximately 93 mg/kg leucine (four to seven times the standard recommended amounts for age for patients with classic MSUD), 1.3 g/kg intact protein, and 0.7 g/kg synthetic protein with leucine levels consistently <200 μmol/L (Table S1).

### 3.2 Impact assessment of p.Thr372Arg variant and selected control variants

**FIGURE 2:**
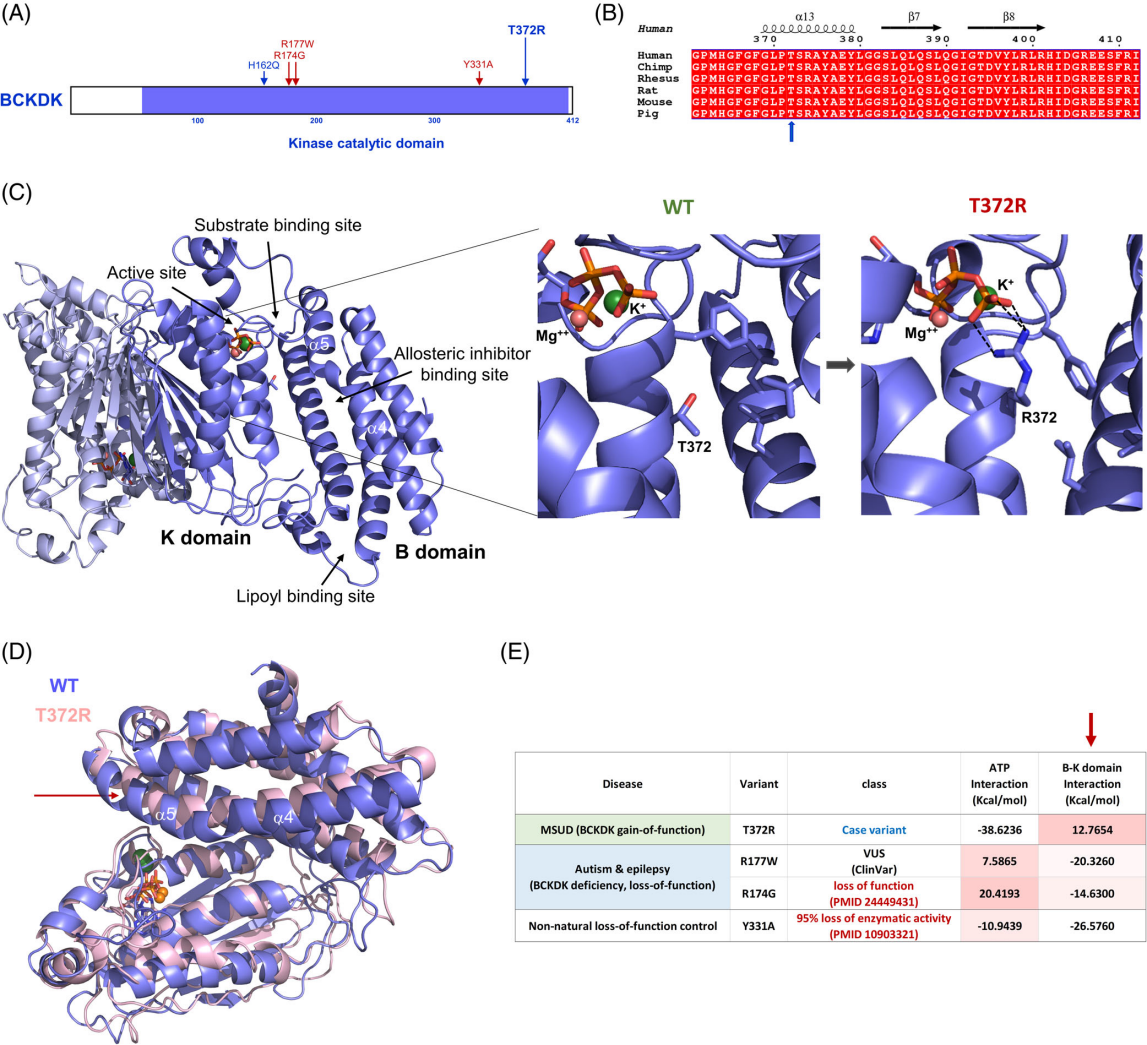
Molecular impact of the p.Thr372Arg variant. (A) Domain structure and the locations of the p.Thr372Arg variant and the control variants in the sequence. (B) Sequence conservation within the region of interest among mammals. The Thr372 residue is indicated by a blue arrow. The sequence alignment of the full‐length protein is shown in Figure S1. (C) A dimeric molecular structure of the BCKDK kinase domain in complex with ATP and the metal ions for impact assessment. The kinase domain is further divided into the active site‐containing K‐domain and the regulatory B‐domain. The key functional sites are also indicated. The close‐up insets show the structural comparison of the wild type and the p.Thr372Arg variant at the end of molecular dynamics simulations. New bond formations help the positioning of the ATP cofactor for a more effective “in‐line” transfer of the phosphoryl group to the substrate. The neighboring hydrophobic residues at the B‐K domain interface are shown as a ball‐and‐stick model. (D) Superposition of the wild type and the p.Thr372Arg variant structures highlighting the outward helical movement of the a4‐a5 central helices by the variant. Loosening up the B‐K domain interface allows optimal substrate binding and presentation for catalysis. (E) Comparison of the alterations in ATP and B‐K domain interaction energies for the p.Thr372Arg case variant and the loss‐of‐function control variants compared to the wild type. The p.Arg174Gly BCKDK deficiency autism variant and p.Try331Ala lab control variant are proven to be loss‐function variants (references indicated) while the p.Arg177Trp BCKDK deficiency autism variant is currently annotated as a variant of uncertain significance (VUS) yet predicted to be a loss‐of‐function variant by our analysis. The B‐K domain interaction energy column is indicated by an arrow. The negative free energies indicate stronger interactions.

The human BCKDK protein is composed of the kinase domain (56–412) and an N‐terminal extension (1–55) whose function remains poorly characterized (Figure 2A). When compared with ortholog *BCKDK* sequences, we observe that the human sequence is conserved throughout mammals (Figures 2B and S1). The kinase domain is further divided into the active site‐containing K‐domain and the four helical bundle regulatory B‐domain (Figure 2C). The B‐domain contains the lipoyl‐binding site and the allosteric inhibitor binding site. The Thr372 residue is located right at the B‐K domain interface (Figure 2C), which is critical for optimal substrate and lipoyl moiety binding.^17^ In addition, proper interaction of these two domains is crucial for the integrity of the nucleotide‐binding site by ordering the nucleotide‐binding pocket surrounding residues between Thr334 and Phe366.^19^ As a control for these analyses, we selected two additional *BCKDK* disease variants at the B‐K domain interface which are associated with autism, epilepsy, and neurological disorders: p.Arg177Trp and p.Arg174Gly. These variants are expected to be loss‐of‐function, though p.Arg177Trp is currently annotated as a VUS in ClinVar.^10^ We also included a control variant, p.Tyr331Ala, which is known to exhibit 95% reduction in its catalytic activity.^26^

Although the sequence‐based pathogenicity predictions for p.Thr372Arg are mixed (Table S2), structure‐ and MD‐based predictions indicate functional alterations by this variant. The B‐K domain interface near the Thr372 residue is mostly hydrophobic (Figure 2C insets) and the substitution to a highly charged bulky residue at this position is expected to disrupt the local structure and inter‐domain communications (Table S2). MD simulation results indicate that the arginine is pushed away from the interface and makes ionic interactions with ATP at the active site (Figure 2C right inset). These new interactions with the γ‐phosphate group help the optimal positioning of the ATP cofactor and thereby a more effective “in‐line” transfer of the phosphoryl group to substrate.^5^ This effect was observed in all 10 replicates of MD simulations, underscoring the reproducibility of the variant behavior over time. Protein folding and stability as well as the principal components of dynamic movements are not significantly altered by this variant (Figure S2).

More importantly, this substitution is expected to cause a loosening up of the B‐K domain interface for better substrate binding and the lipoyl group interactions. It is well known that the allosteric inhibition mechanism involves narrowing of the B‐K domain interface (substrate binding cleft). When the allosteric inhibitor such as α‐ketoisocaproic acid (KIC) binds to an allosteric binding site of BCKDK, it leads to the inward movements of the long helical rod comprising helices α4 and α5, resulting in narrowing the B‐K domain interface, which consequently induces changes in the active site cleft and in the putative lipoyl‐binding site.^17^ The p.Thr372Arg variant causes the opposite, and the α4‐α5 helices move away from the interface resulting in weaker interactions (Figure 2D,E). In contrast, the loss‐of‐function control variants at the B‐K interface all show stronger interactions, thus tightening up the active site cleft and preventing substrates from optimal binding and presentation (Figure 2E). The outward movement of the long α4 and α5 helices in the opposite direction to the allosteric inhibitor bound form is very similar to the movement observed in the other known gain‐of‐function variant p.His162Gln.^16^ Dimeric interactions do not seem to be greatly affected by this variant (Table S2). Taken together, these findings are consistent with the gain‐of‐function activity of the *BCKDK* p.Thr372Arg variant being associated with the biochemical findings of MSUD.

## 4 DISCUSSION

When taken in the context of molecular modeling and reportedly asymptomatic affected family members, our two cases demonstrate that the novel missense variant, p.Thr372Arg, in the *BCKDK* gene may lead to a mild or even benign MSUD biochemical phenotype. The molecular modeling of this variant is consistent with the gain‐of‐function that would result in elevated BCAA, as seen in our patients and positive family members. Family studies revealed multiple clinically well relatives with the p.Thr372Arg variant and the biochemical phenotype of MSUD, consistent with the autosomal dominant inheritance previously reported by Maguolo et al.^11^ To date, there have been no reported metabolic decompensations in the individuals identified with this variant, including the mother and maternal aunt of Patient #1 who have had a combined total of four pregnancies and deliveries. Of note, both Patient #1's mother and Patient #2's father report lower protein intake compared to the typical American diet and high sugar intake in the form of 60–72 ounces of regular soda daily; Patient #1's mother stated, “It's like my body tells me when I'm at my limit for meat,” though Patient #2's father made no similar statements. Both patients have generally maintained plasma leucine levels within MSUD‐treatment ranges despite leucine intake exceeding recommended intake based on GMDI/SERC guidelines.^25^ Prior to the discovery of this variant, Patient #2 had been admitted for precautionary IV dextrose support during multiple intercurrent illnesses per standard MSUD management guidelines. Leucine levels remained within or near typical treatment ranges at or around the time of illness and before IV intervention, drawing into question whether IV support is indeed necessary. Our identification of two healthy children and multiple adults with the p.Thr372Arg variant in the absence of decompensation with stressors suggests that individuals with heterozygous gain‐of‐function variants in *BCKDK* may require less or possibly no acute medical management and dietary manipulation than those with classic MSUD.

Identification of new molecular etiologies for the biochemical phenotype of MSUD has important implications for newborn screening and the diagnostic workup for individuals with elevations in BCAA and alloisoleucine. In the setting of an abnormal newborn screen with elevated BCAA, where classic MSUD may have previously been the presumed clinical diagnosis, molecular testing that includes *BCKDK* and *PPM1K* is important for identifying genotype–phenotype correlations that may tailor medical management and provide early reassurance to parents. As we learn more about this dominant MSUD biochemical phenotype, identifying the parent of origin through sequencing and measuring their BCAAs may aid in elucidating the clinical spectrum. Adults identified as having a *BCKDK* variant following their child's diagnosis will essentially serve as natural history subjects to define the disease spectrum, clarify genotype–phenotype correlations, and illustrate the extent of medical management required.

There is likely an under‐recognition of other mechanisms contributing to the variable clinical phenotypes often seen in rare disease, which may sometimes be explained by a defect in an alternate enzyme or enzyme complex that is typically associated with a specific condition. Modeling does require expertise but is not dependent on cell line or animal model development. We performed both 3D structure‐ and dynamics‐based assessments of the mutational impact of the p.Thr372Arg variant. This type of mechanistic‐based deep variant phenotyping has been proven to improve prediction power beyond sequence information alone and effectively complement the solution‐ and cell‐based functional assays.^27^, ^28^, ^29^ Our results from these comprehensive impact analyses reveal that the p.Thr372Arg variant exhibits gain‐of‐function activities by causing new bond formations with ATP for optimal positioning for an “in‐line” transfer of the phosphoryl group and loosening up the B‐K domain interface for allowing optimal substrate and lipoyl moiety bindings. These findings are consistent with its well‐known catalytic and regulatory mechanisms.^19^ This also supports the concept of targeting this inter‐domain interface for therapeutic intervention in addition to the previously explored target sites, such as the active site and the allosteric regulator binding sites. This is in contrast to the loss‐of‐function of BCKDK, which causes a different biochemical and clinical phenotype as discussed earlier.

As novel variants are discovered, this type of modeling can be helpful to clarify their functional consequences to better understand clinical phenotype, inform patient care, and elucidate new potential therapeutic approaches. Until recently, management of MSUD relied on nutritional management alone, though liver transplantation has now been completed in many individuals.^30^ While transplants allow individuals to liberalize their diet and prevent metabolic crises, it does not appear to reverse the neurologic sequelae associated with MSUD.^31^ The medication phenylbutyrate, as well as gene therapies, are under investigation for clinical application in classic MSUD.^31^, ^32^, ^33^, ^34^ However, with a better understanding that gain‐of‐function variants in *BCKDK* lead to impaired BCAA metabolism, could a kinase inhibitor meaningfully increase activity in branched‐chain metabolism for individuals that have some residual enzyme activity in the BCKD complex? Given that loss‐of‐function of the BCKDK enzyme leads to a different phenotype (developmental delay, microcephaly, autism spectrum disorder, seizures, and biochemical evidence of low BCAA), a cautious approach would be required to achieve the delicate balance needed to achieve a therapeutic outcome.^35^ Overall, this new knowledge will benefit the increasing number of patients carrying similar variants by shedding light on new molecular mechanisms for an MSUD biochemical phenotype and potentially leading to new avenues for therapy.

Lastly, it is scientifically appropriate to discuss potential pitfalls and alternative interpretations of our results. For instance, could we have missed a classic recessive diagnosis of MSUD with standard sequencing as performed in these cases? However, with the biochemical and genetic evidence and unremarkable health history identified in multiple family members, this would be an unlikely scenario. Second, as we only present two families with this variant, there could be other genetic/environmental modifiers impacting their seemingly asymptomatic presentation. Additionally, while this modeling technique does look at the 3D structure and activity of the enzyme, it is not examined directly within the cell as a cell or animal model would be, and thus could miss other interferences that could impact kinase function.

## 5 CONCLUSION

MSUD is a potentially severe, life‐threatening protein metabolism disorder, but—like other metabolic disorders—milder and even asymptomatic clinical phenotypes have emerged due to the complexity of catabolism. The biochemical spectrum of branched‐chain amino acid metabolism may represent a range of severity and underlying genotypes. The gene *BCKDK*, encoding for branched‐chain ketoacid dehydrogenase kinase, has been found to modulate BCAA metabolism; first in individuals with loss‐of‐function variants, low BCAAs, and a treatable neurodevelopmental phenotype, and more recently a single case report has identified a family with a gain‐of‐function variant, elevated BCAAs, and a mild or possibly benign biochemical phenotype of MSUD. We report two unrelated patients detected on newborn screening who were found to have the same novel *BCKDK* variant, a biochemical phenotype consistent with MSUD, unremarkable clinical course, and healthy adult family members with similar findings. The molecular modeling of the variant revealed an explanation for the biochemical presentation. Our report highlights the utility of family studies and molecular modeling to evaluate the impact of novel variants in metabolism. This discovery has significant clinical implications, mainly suggesting that little or no clinical intervention may be needed for patients with a biochemical phenotype consistent with MSUD due to heterozygous gain‐of‐function *BCKDK* variants.

## AUTHOR CONTRIBUTIONS

**Emily Singh**: contributed to patient care, family member testing, data collection, and preparation of the manuscript. **Young‐In Chi**: contributed to 3D‐modeling and preparation of the manuscript. **Jessica Kopesky**: contributed to patient care, data collection, and the preparation of the manuscript. **Michael Zimmerman**: contributed to 3D‐modeling and review of the manuscript. **Raul Urrutia**: contributed to 3D‐modeling and review of the manuscript. **Donald Basel**: contributed to patient care and review of the manuscript. **Jessica Scott Schwoerer**: contributed to patient care, data collection, and preparation of the manuscript.

## FUNDING INFORMATION

Family studies were funded by the Medical College of Wisconsin Undiagnosed and Rare Disease Program.

## CONFLICT OF INTEREST STATEMENT

The authors declare no conflicts of interest.

## INFORMED CONSENT

All procedures followed were in accordance with the ethical standards of the committee responsible for human experimentation (institutional and national) and with the Helsinki Declaration of 1975, as revised in 2000 (5). No formal IRB or ethics approval was needed for the study. Informed consent was obtained from all patients for being included in the study. Consent for publication was obtained from the parent of each patient in the case report using the BMJ consent form.

## Supporting information


**Table S1.** Detailed diet history with additional biochemical results.

**Table S2.** Spreadsheet of individual damaging scores by each analysis. The damaging metrics from each protein layer are indicated by different colors: sequence‐ (2D, blue), structure‐ (3D, red), and MD‐based (4D, purple). In this table, the values are shown for the p.Thre372Arg case variant and the control variants. Although 2D‐based pathogenicity predictions for the p.Thr372Arg variant are mixed, the 3D‐, and 4D‐based analyses suggest structural perturbations, enhanced ATP interactions, and reduced B‐K domain interactions.

**Figure S1.** A sequence alignment of *BCKDK* from selected mammals. Secondary structure elements are shown at the top of the sequence alignment. The most variable region is found at the N‐terminal extension and the kinase domain sequences are highly conserved among mammals.

**Figure S2.** Porcupine plot of trajectories representing the essential dynamic motions of the wild type and the p.Thr372Arg variant in the first major principal component (PC1) during MD simulation. They both show similar directional movements. The red arrows represent the magnitude and the direction of dynamic motions.

## Data Availability

The data that supports the findings of this study are available in the supplementary material of this article.
