# Supplementary material for: Computational structural genomics and clinical evidence suggest BCKDK gain‐of‐function may cause a potentially asymptomatic maple syrup urine disease phenotype
Source: JIMD Rep. 2024 Apr 8;65(3):144–55. doi: 10.1002/jmd2.12419 (PMC11078707; doi:10.1002/jmd2.12419)
Supplement: Supplementary file 1 — Table S1. Detailed diet history with additional biochemical results. [file JMD2-65-144-s001.pdf]

Supplementary Table S2: Detailed diet history with additional biochemical results

	**Prescribed Leu mg/day (mg/kg)**	**Prescribed Iso mg/day (mg/kg)**	**Prescribed Val mg/day (mg/kg)**	**Prescribed T Pro g/day (g/kg)**	**Corresponding Plasma Leu/Iso/Val (µmol/L)**	**Major diet components/ Clinical notes**
**Patient #1**						
Baseline estimated intake (Before intervention)	433 (397)[a]	257 (236)	297 (272)	4.4 (4.03)	420/307/505	DHM + HMF
Initial diet implementation	182 (161)[a]	130 (115)	297 (272)	2.8 (2.48)	224/91/204	DHM + IVA EY
Most restrictive prescription	144 (115)[a]	152 (121)	173 (137)	5.02 (4)	30/117/304	DHM + IVA EY + MSD AA
Maintenance prescription before challenge implemented	1036 (145)[b]	534 (75)	594 (83)	18.32 (2.6)	152/94/113	Sim Neo + MSUD EY + foods
Leu challenge (ongoing)[c]	1119 (132)	534 (69)	670 (79)	21.2 (2.5)	169/98/130	Sim Neo + WM + MSUD EY + foods
	1128 (119)	599 (63)	713 (75)	21.7 (2.3)	179/110/132	
**Patient #2**						
Baseline estimated intake (Before intervention)	936-1540 (255-420)[a]	552-916 (150-250)	621-926 (169-252)	10.2-14.7 (2.8-4)	360/180/489	BF + Enf Gen
Initial diet implementation/ Most restrictive prescription	232-344 (53-79)[a]	304-368 (70-84)	344-400 (79-92)	13.6-14.4 (3.1-3.3)	14/227/568	BF+ HM + Enf Gen + MSUD EY + IVA EY
Maintenance prescription before challenge implemented	959[d]	795	981	34	124/170/243	WM + IVA EY + MSD Ess + foods
Leu challenge (ongoing)[d]	1031	900	1116	37 (1.9)	107/131/171	WM + IVA EY + MSD Ess + foods
	1258	968	1203	38.6 (2)	132/142/217	
	1467	1087	1355	41.6 (2.1)	172/154/210	
	1646	1187	1483	41.4 (2.1)	171/147/170	
	1797	1271	1589	38.3 (2)	165/212/263	
	1977	1349	1688	39.5 (2)	197/115/174	

Abbreviations: Leu=leucine; Iso=isoleucine; Val=valine; TPro=Total Protein; DHM=Donor Human Milk; HMF=Human Milk Fortifier; IVA EY=IVA Anamix Early Years™; MSD AA=Complex MSD Amino Acid Blend™; Sim Neo=Similac NeosureTM, powder; MSUD EY=MSUD Anamix Early Years™; BF=At chest breastfeeding; Enf Gen=Enfamil Gentlease™; HM=human milk; WM=whole milk; MSD Ess=Complex Essentials MSD™

Standard recommended intake ranges of leu for classic MSUD from GMDI/SERC guidelines: [a]0-3mos: 60-100mg/kg [b]6-12mos: 35-70mg/kg [c]12-48mos: 25-55mg/kg [d]≥48mos: 275-500mg/day
