# Supplementary material for: Computational structural genomics and clinical evidence suggest BCKDK gain‐of‐function may cause a potentially asymptomatic maple syrup urine disease phenotype
Source: JIMD Rep. 2024 Apr 8;65(3):144–55. doi: 10.1002/jmd2.12419 (PMC11078707; doi:10.1002/jmd2.12419)
Supplement: Supplementary file 2 — Table S2. Spreadsheet of individual damaging scores by each analysis. The damaging metrics from each protein layer are indicated by different colors: sequence‐ (2D, blue), structure‐ (3D, red), and MD‐based (4D, purple). In this table, the values are shown for the p.Thre372Arg case variant and the control variants. Although 2D‐based pathogenicity predictions for the p.Thr372Arg variant are mixed, the 3D‐, and 4D‐based analyses suggest structural perturbations, enhanced ATP interactions, and reduced B‐K domain interactions. [file JMD2-65-144-s004.pdf]

Disease	Variant	class	Note	SNPsGo	Mutation Assessor	REVEL	CADD	PolyPhen2	Missense 3D	Rhapsody	FoldX	Stability Energy	Global Perturb. (Å)	Local Perturb. (Å)	Global Frustration	ATPinteraction (kcal/mol)	BKdomainInteraction (kcal/mol)	DimerInteraction (kcal/mol)
MSUD (BCKDK gain-of-function)	**T372R**	**case variant**	B-K domain interface (hydrophobic environment)	0.468	1.04	0.327	4.0844	0.963	0	0.813	-0.4814	0.47	0.0898	0.1795	13.170	-38.6236	12.7654	6.7371
	H162Q	gain of function (PMID: 35205278)	right at the allosteric inhibitor binding site near the H4-H5 kink	0.816	4.08	0.685	3.3161	0.997	1	0.852	0.9824	-1.15	0.0576	0.1404	7.342	8.7405	-11.0910	8.0385
Autism, epilepsy, developmental & neurological	R177W	VUS (ClinVar)	B-K domain interace, near the ATP binding pocket	0.483	3.395	0.428	4.22	0.893	0	0.665	1.7279	-0.52	0.0722	0.1846	14.529	7.5865	-20.3260	23.792
	R174G	loss of function (PMID: 24449431)	B-K domain interface	0.227	2.19	0.16	4.8973	0.928	0	0.657	1.4191	1.97	0.0530	0.1399	11.416	20.4193	-14.6300	-15.0708
Non-natural loss-of-function control	Y331A	95% loss of enzymatic activity (PMID: 10903321)	B-K domain interface	0.583	N/A	N/A	N/A	1	3	0.929	4.8613	3.97	0.0370	0.0859	6.980	-10.9439	-26.5760	-4.1584
