# Supplementary figures and images for: Computational structural genomics and clinical evidence suggest BCKDK gain‐of‐function may cause a potentially asymptomatic maple syrup urine disease phenotype
Source: JIMD Rep. 2024 Apr 8;65(3):144–55. doi: 10.1002/jmd2.12419 (PMC11078707; doi:10.1002/jmd2.12419)

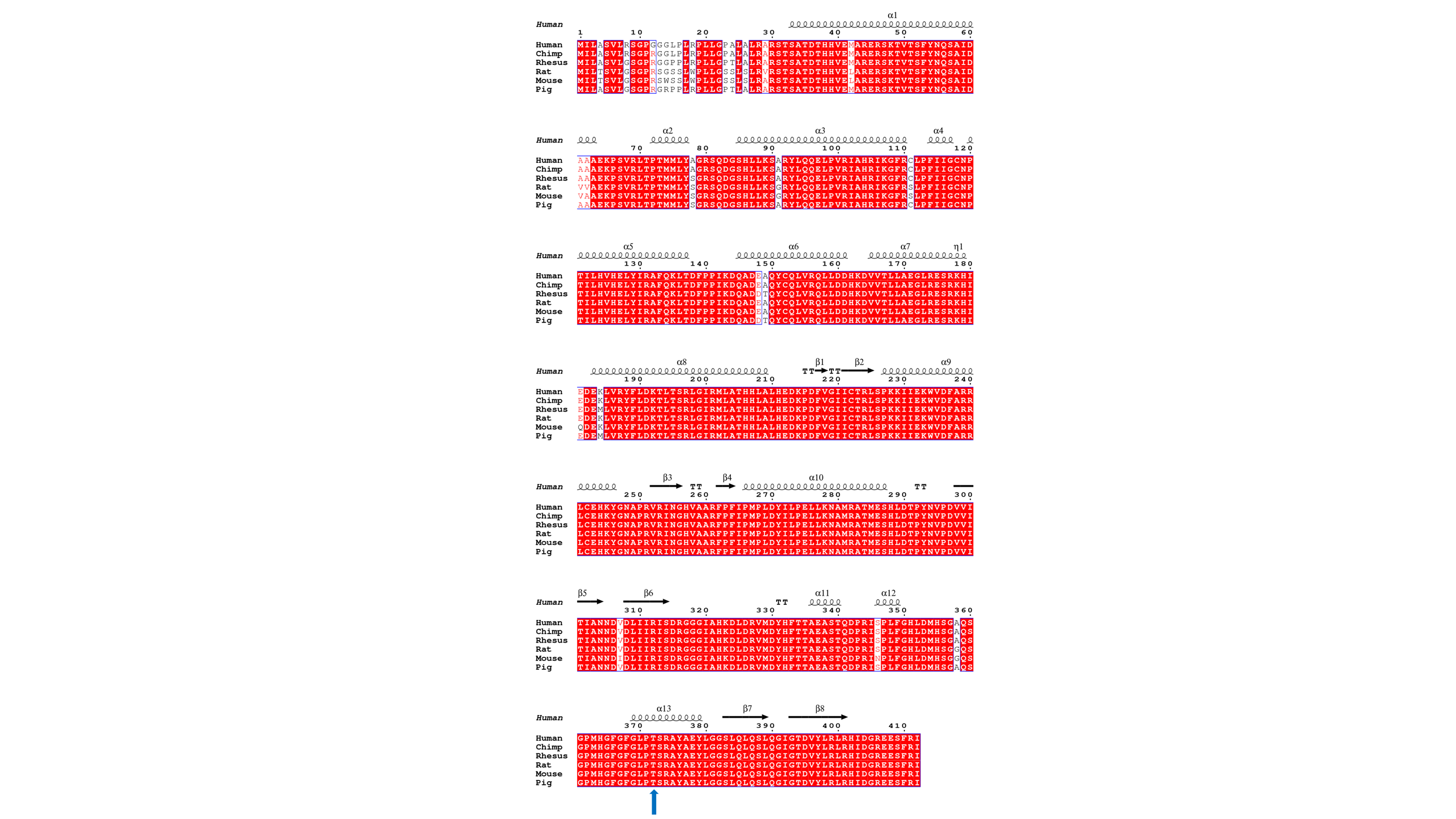

Supplement: Supplementary file 3 — Figure S1. A sequence alignment of BCKDK from selected mammals. Secondary structure elements are shown at the top of the sequence alignment. The most variable region is found at the N‐terminal extension and the kinase domain sequences are highly conserved among mammals. [file JMD2-65-144-s003.tif]

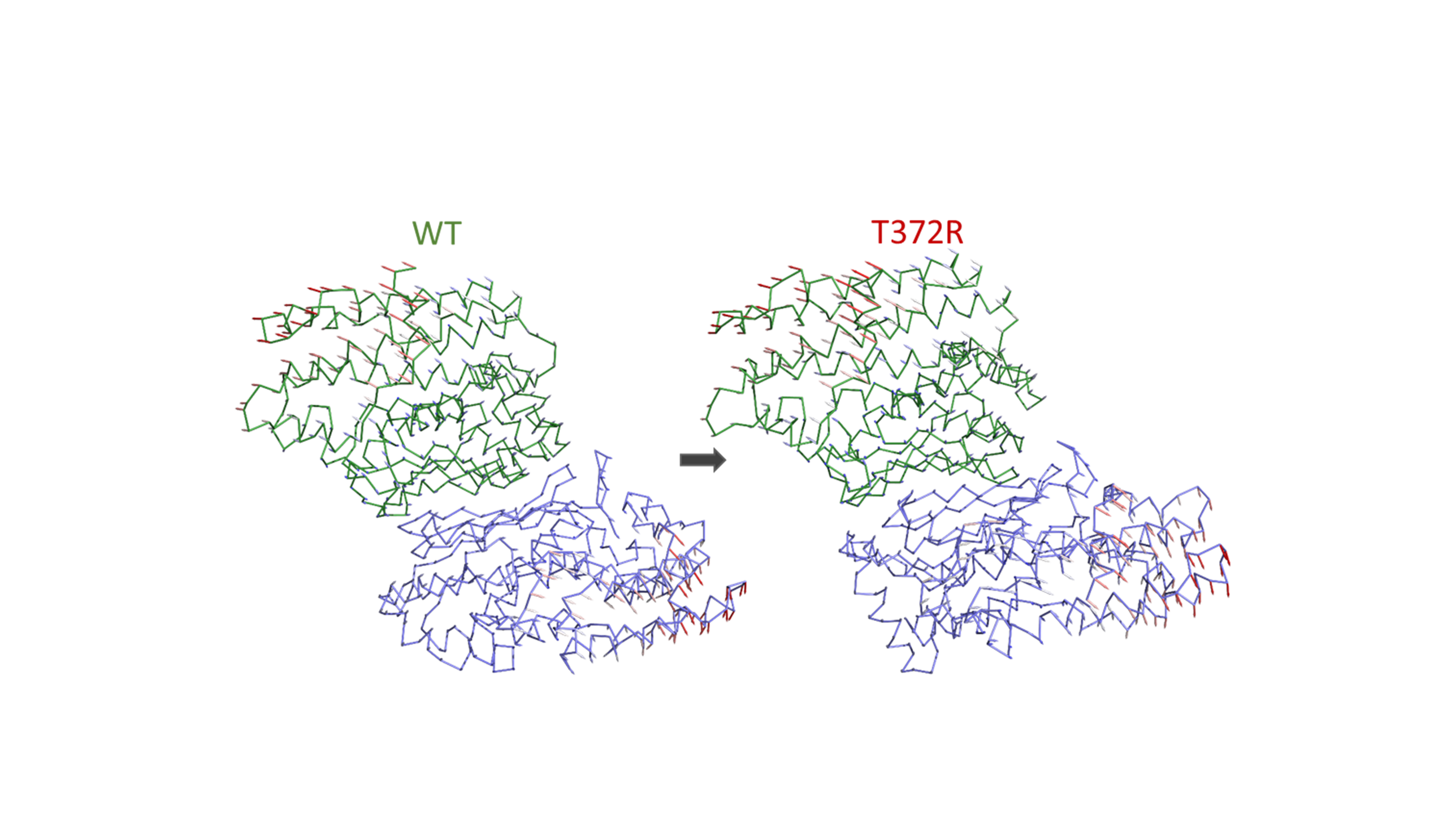

Supplement: Supplementary file 4 — Figure S2. Porcupine plot of trajectories representing the essential dynamic motions of the wild type and the p.Thr372Arg variant in the first major principal component (PC1) during MD simulation. They both show similar directional movements. The red arrows represent the magnitude and the direction of dynamic motions. [file JMD2-65-144-s002.tif]
